# Influenza C infections in Western Australia and Victoria from 2008 to 2014

**DOI:** 10.1111/irv.12402

**Published:** 2016-07-23

**Authors:** Lauren Jelley, Avram Levy, Yi‐Mo Deng, Natalie Spirason, Jurissa Lang, Iwona Buettner, Julian Druce, Chris Blyth, Paul Effler, David Smith, Ian G. Barr

**Affiliations:** ^1^ WHO Collaborating Centre for Reference and ResearchMelbourneVic.Australia; ^2^ PathWest Laboratory Medicine WANedlandsWAAustralia; ^3^ School of Pathology and Laboratory MedicineUniversity of Western AustraliaPerthWAAustralia; ^4^ Victorian Infectious Disease Reference LaboratoryMelbourneVic.Australia; ^5^ School of Paediatrics and Child HealthUniversity of Western AustraliaPerthWAAustralia; ^6^ Princess Margaret Hospital for ChildrenSubiaco, PerthWAAustralia; ^7^ Wesfarmers Centre for Vaccines and infectious DiseasesTelethon Kids InstitutePerthWAAustralia; ^8^ Communicable Disease Control DirectorateDepartment of HealthShenton ParkWAAustralia; ^9^ Department of Microbiology and ImmunologyUniversity of MelbourneMelbourneVic.Australia

**Keywords:** Australia, children, influenza C, respiratory disease, viruses

## Abstract

**Background:**

Influenza C is usually considered a minor cause of respiratory illness in humans with many infections being asymptomatic or clinically mild. Large outbreaks can occur periodically resulting in significant morbidity.

**Objectives:**

This study aimed at analyzing the available influenza C clinical samples from two widely separated states of Australia, collected over a 7‐year period and to compare them with influenza C viruses detected in other parts of the world in recent years.

**Patients/Methods:**

Between 2008 and 2014, 86 respiratory samples that were influenza C positive were collected from subjects with influenza‐like illness living in the states of Victoria and Western Australia. A battery of other respiratory viruses were also tested for in these influenza C‐positive samples. Virus isolation was attempted on all of these clinical samples, and gene sequencing was performed on all influenza C‐positive cultures.

**Results and conclusions:**

Detections of influenza C in respiratory samples were sporadic in most years studied, but higher rates of infection occurred in 2012 and 2014. Many of the patients with influenza C had coinfections with other respiratory pathogens. Phylogenetic analysis of the full‐length hemagglutinin–esterase–fusion (HE) gene found that most of the viruses grouped in the C/Sao Paulo/378/82 clade with the remainder grouping in the C/Kanagawa/1/76 clade.

## 1 Introduction

Influenza C is often ignored compared with the other human pathogens in the Orthomyxoviridae family (influenza A and influenza B). With respect to other influenza types, it causes a milder disease with fewer complications and, as it has not been included in routine testing, there is little new information being generated about its impact. In etiologic studies of respiratory illnesses that have included influenza C testing, it usually accounts for a low proportion of acute respiratory pathogens identified. For example, a Canadian study^1^ found 2.32% of respiratory samples tested from children identified influenza C, while a study in Japan spanning 18 years^2^ (1996–2013) found influenza C in 0.43%–1.73% of samples from children; this result was similar to a Spanish study that reported influenza C in 0.7% of children's samples.^3^ Low rates were reported in a retrospective study Scottish study that screened 3300 respiratory samples collected from children and adults during the period from August 2006 to June 2008 with only six positive influenza C‐positive samples (0.2%) identified (with 4/6 from children ≤2 year) compared to 3.2% influenza A detections and 0.9% influenza B detections.^4^ Higher rates of influenza C infection have been reported in a Nigerian study, where 4.8% of the respiratory samples from children were positive for influenza C^5^ and Finnish study among young adult male military recruits,^6^ in which influenza C was identified in 4.2% of all samples. Influenza C can also be identified in a significant proportion of children hospitalized with lower respiratory tract infections, as reported by Shimizu et al.^7^ 2015, who found approximately 10% of children had influenza C present in samples collected from four Japanese hospitals during 2009–2010. Often these cases of influenza C also have other viruses or bacteria detected in the specimen making it difficult to attribute the contribution of influenza C infections to the clinical illness manifestations.^5^, ^8^ In contrast to most viral etiology studies, serological studies often find high seroprevalence rates in the community which rise rapidly up to 10 years of age to around 50%–60% of the population, indicating that widespread transmission has occurred among children in many different countries.^9^ Unlike influenza A and B infections where early treatment with neuraminidase inhibitor drugs can be effective, there are no effective antiviral treatments for influenza C.

This study analyzed influenza C viruses detected in respiratory samples collected from two influenza illness surveillance programs operating in the state of Western Australia (WA) from 2001 to 2014: one covering patients of all ages presenting to general practitioners with an influenza‐like illness (ILI), and the other covering young children presenting with a respiratory illness to a metropolitan pediatric hospital emergency department or a general practitioner. These were compared with influenza C viruses detected from routine screening of respiratory samples from Melbourne, Victoria from 2011 to 2014. This report is the first to isolate and characterize (including sequencing) influenza C viruses collected in Australia.

## 2 Materials and Methods

### 2.1 Patients and specimens

Human influenza C is not part of routine respiratory virus testing in most Australian laboratories. From 2001 to 2011, 16 of 9463 (0.17%) human respiratory samples received from the WA sentinel General Practitioner (GP) influenza surveillance network known as SPN(WA)^10^ tested positive for influenza C by tissue culture or real‐time PCR and 63 samples from 4034 samples (1.6%) received between 2008 and 2014 from the Western Australian Influenza Vaccine Effectiveness (WAIVE) study^11^ tested positive for influenza C by real‐time PCR. Testing for all WA respiratory samples was performed at the PathWest Laboratory Medicine WA in Perth, WA. The WAIVE study collected nasopharyngeal aspirates from children presenting to the Princess Margaret Children's Hospital emergency department during the influenza season (usually June/July–October), whereas SPNWA collected nose and throat swabs from patients presenting to GP clinics with ILI year‐round. Testing for various respiratory pathogens varied between 2002 and 2014 as new tests were developed, but in general, samples were tested for influenza A, B, and C, influenza subtypes, human metapneumovirus, respiratory syncytial virus A and B, and parainfluenza types 1–3 by PCR. WAIVE testing also included additional PCR tests for parainfluenza type 4, *Bordetella pertussis*, human bocavirus, coronaviruses HKU1, OC43, 229E and NL63, enteroviruses (including rhinovirus) and human adenoviruses. From 2012 onwards, samples from SPN(WA) were no longer tested for influenza C due to the low detection rates. Further influenza C‐positive respiratory samples were obtained from VIDRL, Melbourne, Victoria, during the period 2011–2014 when samples were tested for the presence of a panel of respiratory viruses by real‐time PCR.

### 2.2 Virus isolation in cell culture

Isolation of influenza C viruses was performed using Madin–Darby canine kidney (MDCK) cells (ATCC CCL‐34) using maintenance media (DMEM Coon's Basal Media containing sodium bicarbonate (3%) with the addition of 2 mmol/L glutamine, 1% non‐essential amino acids, 0.05% NaHCO3, 0.02 mol/L HEPES, 4% penicillin and streptomycin, 2 μg/mL amphotericin B and 4 μg/mL trypsin). Samples were incubated for up to 5 days at 33°C without CO_2_, and virus growth was quantified by determining the hemagglutination titer (HA) with 0.5% fowl and turkey RBC.^8^, ^12^

### 2.3 Real‐time (RT‐PCR) assay

Prior to sequencing or culturing, due to the age of some of the clinical samples, a real‐time PCR assay was designed and run so that samples could be retested at the WHO Centre to confirm that viral RNA was still detectable. Primers and a minor groove binding probe were designed using an alignment of influenza C hemagglutinin–esterase–fusion (HE) gene sequences from GenBank. Sequences were downloaded and aligned using the Megalign program of DNAStar (Madison, WI, Australia), and HE primers and probes were obtained (GeneWorks, Adelaide, SA, Australia) and used for the real‐time PCR of clinical samples and also for the confirmation of influenza C virus isolates (Table 1). For the real‐time PCR assay, RNA was extracted from virus isolates and original clinical samples using the QIAGEN QIAamp Viral RNA mini kit (Qiagen, Australia) according to the manufacturer's instructions using an elution volume of 60 μL of viral RNA in water. A one‐step RT‐PCR assay using the SensiFAST Probe Lo‐ROX one‐step kit (Bioline, Melbourne, Australia) was performed according to the manufacturer's instructions using 4ul of viral RNA with 16ul of mastermix, which included 0.2ul of each forward primer and reverse primer, and probe (Applied Biosystems, Australia). For HE gene detection, samples were then run on an ABI‐7500 fast real‐time PCR instrument (Applied Biosystems, Melbourne, Australia) with a program set at one cycle of 45° for 10 minutes, one cycle of 95° for 2 minutes and an amplification step of 40 cycles of a denaturation step of 95° for 5 seconds and an annealing and extension step of 60° for 30 seconds. Cycle threshold (Ct) values were determined automatically.

**Table 1:** Primer and probe sequence information used in the real‐time assay and to sequence the HE gene

Primer/Probe	Assay	Sequence
HE RT Forward Primer	Real‐time assay	GCATCTTGTGGCTTCTTG
HE RT Reverse Primer	Real‐time assay	TRGGAGAGCTTCTTACTG
HE MGB Probe	Real‐time assay	CACAGGGAATTCTGGAGACAC
HE Forward primer I	PCR	TGTAAAACGACGGCCAGTAGCAGAAGCAG
HE Reverse primer I	PCR	CAGGAAACAGCTATGACCGAATCATAGATCAC
HE forward primer II	PCR	TGTAAAACGACGGCCAGTAAAAGAGGATGTG
HE reverse primer II	PCR	CAGGAAACAGCTATGACCCTTCAGATCTTGCA
HE forward primer III	PCR	TGTAAAACGACGGCCAGTATGAGCAAGCCATC
HE reverse primer III	PCR	CAGGAAACAGCTATGACCAGCAGTAGCAAG
13F primer	Sequencing	TGTAAAACGACGGCCAGT
M13R primer	Sequencing	CAGGAAACAGCTATGACC

### 2.4 Gene sequencing and phylogenetic analysis

Purified RNA extracted from influenza C‐positive samples was amplified using the BIOLINE MYTAQ one‐step RT‐PCR kit (Bioline, Australia) using in‐house‐designed HE gene‐specific primers supplied by Bioneer (Melbourne, Australia; Table 1). RT‐PCR was performed in a 50 μL reaction volume as follows: 50°C 30 minute, 94°C 2 minute, then 40 cycles of 94°C 30 seconds, 55°C 30 seconds, 68°C 30 seconds, then a final extension at 68°C for 1 minute. RT‐PCR products were purified by ExoSAP‐IT (GE Healthcare, Sydney, Australia) and then subjected to sequencing reactions using either M13F or M13R primers with Big Dye Terminator Reaction Mix (Applied Biosystems, Australia). The reaction products were purified by Big Dye XTerminator Purification Kit (Applied Biosystems, Australia) and run on ABI 3500XL sequencer. Sequence assembly was performed using Seqman Pro Module of DNAStar (Madison, WI, Australia), and phylogenetic analysis was carried out using Geneious v9.0.4 (Biomatters, Auckland, New Zealand). A Phylogenetic tree and the bootstrap supports for the HE data set were estimated using the best‐fit evolutionary model and the maximum likelihood (ML) method in PhyML, v3.0, software.^13^ Other representative influenza C viruses’ HE sequences were downloaded from GenBank, including the influenza C viruses that make up the antigenically and phylo‐genetically distinct reference groups: C/Taylor/1233.47, C/Aichi/1/81, C/Sao Paulo/378/82, C/Kanagawa/1/76, Yamagata/26/81 and C/Mississippi/80.^2^, ^8^ Bootstrapping estimates (%) were generated after running 1000 replicates.

## 3 Results

### 3.1 Patient details and initial testing

For the 16 influenza C‐positive samples from the SPN(WA) GP surveillance, the age range was 1–40 years with the median age of 22 years (25% male, 75% female), while for the 63 influenza C‐positive WAIVE samples the age range was 0.6–5.1 years with a median age of 1.7 year (46% male, 54% female). Note that the WAIVE program only recruits children aged between 6 months and 59 months who attended the Emergency Department or were admitted to the Princess Margaret Hospital, Perth (WA) with an ILI (defined by the presence or history of fever and a respiratory symptom). This study group was therefore made up exclusively of young children and they had higher rates of influenza C than the SPN(WA) group (Table 1).

During the WAIVE study, the overall prevalence of influenza C cases was 1.6% and varied from 0% (2009) up to 5.8% (2014) of the cases tested, while the annual incidence was lower in SPN(WA) samples with an overall prevalence of 0.17% with a range of 0% (2006) to 0.8% (2008) of samples tested being positive for influenza C (Table 2). Over the same study periods, the WAIVE respiratory samples had the following ranges of percentage positive samples for influenza A: 7.3%–21.9%, influenza B: 0%–15.2% and RSV 11.7%–21.4%, while the SPNWA samples had ranges for influenza A: 3.1%–35.4%, influenza B: 0.3%–19.5%, and RSV 0.5%–8.0%. For the WAIVE 2014 influenza C‐positive clinical samples, there were generally coinfections present with other viral pathogens detected (one other virus = 12 cases, two other viruses = 10 cases, three other viruses = 3 cases, and four other viruses = 1 case) and only 4 (13%) of samples had influenza C infections alone detected (Table 3). The most common multiple infections along with influenza C were with human enteroviruses/rhinoviruses (13 samples), influenza A (seven samples; four with influenza A H1N1pdm09 and three with influenza A(H3N2)) followed by RSV (six samples) and adenovirus (six samples) (Table 3). No clinical details (hospital/ICU admission data) of cases were available from the SPN(WA) study; however, hospital admission data (no ICU data) were available for the WAIVE study. For the entire WAIVE study cohort, 678/4034 (16.8%) of cases were admitted to hospital compared with 7/63 (11.1%) of cases admitted where influenza C was detected. In the WAIVE 2014 study, all of the influenza C cases were obtained from residents of the Perth metropolitan region, with the majority of cases occurring across the Northern and Southern suburbs (Figure S1), but there was no evidence of substantial clustering by residential postcode or place of admission. The non‐metropolitan cases were from Broome in the Kimberley region some 2240 km north‐east of Perth, and from Kalgoorlie in the Goldfields approximately 600 km east of Perth. The seven *ad hoc* Victorian influenza C‐positive samples (one from 2011, two from 2012, and four from 2014) had an age range from 0.4 year to 54.5 year with two samples (out of six where the age was known) <5 year of age.

**Table 2:** Influenza C viruses detected in WA influenza surveillance studies

Year	Samples tested	SPNWA		Samples tested	WAIVE		Samples sent to WHO CC
		Flu C # cases	Flu C % positive		Flu C # cases	Flu C % positive	
2001^a^	123	0	0	–	–	–	0
2002^a^	194	1	0.5	–	–	–	1
2003^a^	278	0	0	–	–	–	0
2004	191	2	0.7	–	–	–	0
2005	266	0	0	–	–	–	0
2006	174	0	0	–	–	–	0
2007	594	2	0.3	–	–	–	2
2008	771	6	0.8	757	1	0.1	4
2009	1425	1	0.1	604	0	0	1
2010	751	3	0.4	272	11	4.0	8
2011	754	1	0.1	608	0	0	1
2012	1647	–	–	747	19	2.5	13
2013	1068	–	–	511	1	0.2	0
2014	1129	–	–	538	31	5.8	31
Total	9463	16	0.2	4034	63	1.6	61

^a^Tissue culture only, no PCR detection; Note that WAIVE did not commence until 2008 and respiratory samples from SPN(WA) have not been tested for influenza C since December 31, 2011.

**Table 3:** Respiratory samples containing Influenza C and other viruses from Perth in 2014

Designation	Age (Y)	Sex	Sample date *D/M/Y*	Infl. C isolate	HE Seq isolate	Viruses apart from influenza C detected at initial sample test
C/PERTH/1/2014	3.3	F	18/07/2014	Y	Y	RSVA, EV/RV
C/PERTH/2/2014	2.7	M	20/07/2014	Y	Y	Influenza C only
C/PERTH/3/2014	1.0	M	25/07/2014	N	N	RSVA
C/PERTH/4/2014	2.8	M	26/07/2014	N^a^	N	Influenza A/H1N109
C/PERTH/5/2014	1.5	F	6/08/2014	Y	Y	RSVA, hCoV(HKU1), hBoV
C/PERTH/6/2014	0.8	F	6/08/2014	N	N	Influenza C only
C/PERTH/7/2014	0.8	F	8/08/2014	N	N	hBoV
C/PERTH/8/2014	1.8	M	31/07/2014	Y	Y	I hCoV(OC43)
C/PERTH/9/2014	3.5	F	17/08/2014	Y	Y	I Adeno
C/PERTH/10/2014	5.2	M	17/08/2014	Y	Y	EV/RV
C/PERTH/11/2014	1.9	M	16/08/2014	Y^a^	P**	Influenza A/H1N109, EV/RV
C/PERTH/12/2014	2.4	M	18/08/2014	Y	Y	, RSVA
C/PERTH/13/2014	1.6	F	21/08/2014	N	N	PIV3, RSVA, EV/RV
C/PERTH/14/2014	1.4	F	12/08/2014	N	N	PIV3, hBoV, EV/RV
C/PERTH/15/2014	2.8	M	13/08/2014	Y	Y	Influenza C only
C/PERTH/16/2014	1.3	F	13/08/2014	N	N	Influenza A/H3N2, Adeno
C/PERTH/17/2014	0.8	M	14/08/2014	Y	Y	EV/RV
C/PERTH/18/2014	1.4	F	14/08/2014	N	N	, EV/RV, Adeno
C/PERTH/19/2014	1.4	F	23/08/2014	Y	Y	PIV4A, hCoV(OC43), hBoV, EV/RV
C/PERTH/20/2014	3.6	M	24/08/2014	N^a^	N	Influenza A/H1N109
C/PERTH/21/2014	3.8	F	24/08/2014	N	N	Adeno
C/PERTH/22/2014	0.6	M	25/08/2014	N	N	EV/RV
C/PERTH/23/2014	2.8	F	26/08/2014	Y	Y	Influenza C only
C/PERTH/24/2014	2.5	F	25/08/2014	N^a^	N	Influenza A/H1N109 hMPV
C/PERTH/25/2014	0.7	F	9/09/2014	N	N	RSVA, Adeno
C/PERTH/26/2014	1.7	M	13/09/2014	Y	Y	PIV3, hCoV(OC43), EV/RV
C/PERTH/27/2014	1.7	F	17/08/2014	N	N	I hMPV, EV/RV
C/PERTH/28/2014	1.8	M	10/08/2014	N	N	hMPV, EV/RV
C/PERTH/29/2014	1.3	F	2/08/2014	N	N	Influenza A/H3N2, Adeno
C/PERTH/30/2014	1.8	F	2/09/2014	N^a^	N	Influenza A/H3N2
C/PERTH/31/2014	1.5	M	3/10/2014	Y	Y	hBoV, EV/RV

^a^Influenza A isolated alone or as well as influenza C; P** = Partial HE sequence obtained.

Virus isolation was attempted for all influenza C‐positive clinical samples received at the Centre. Overall, the isolation rate for influenza C viruses was successful for 35/68 samples, 52% (with 16/30; 53% for Perth samples 2002–2012, 6/7; 86% for Victorian samples and 13/31; 42% for the 2014 Perth samples; Table 3) based on HA of turkey RBC (fowl RBC gave virtually identical HA titers) and confirmed by real‐time PCR detection of the HE gene. HA titers ranged from 1 to 128, and the real‐time PCR cycle threshold (Ct) value for these HA‐positive cell isolates (using the HE primers and probe as outlined in Table 1) ranged from 11.5 to 22.05.

### 3.2 Sequencing and phylogenetic analysis

**Figure 1:**
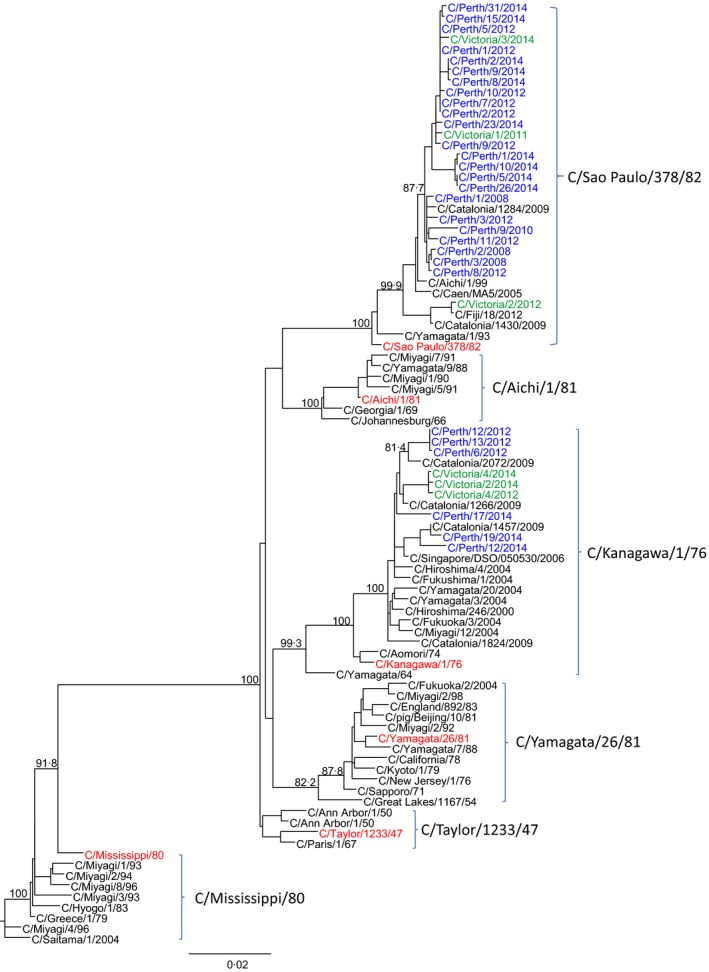
Phylogenetic analysis of the HE genes from Australian and reference influenza C viruses using the maximum likelihood method with bootstrap values indicated on the branches (n = 1000 replicates). GISAID accession numbers are contained in Table S1

Hemagglutinin–esterase–fusion gene sequence was obtained for all samples that yielded influenza C virus isolates. Thirty‐five influenza C full HE sequences (28 from Perth/WA and seven from Victoria) and one partial HE sequence from Perth were obtained from the virus isolates. These sequences were compared with publically available HE sequences from reference viruses including those that represent the six antigenically distinct influenza C groups from recently circulating influenza C viruses (Fig. 1). The final data set contained 89 HE genes. The maximum likelihood phylogenetic analysis of the HE virus genes from the Australian influenza C isolates showed that they fell into only two of the six reference clades,^2^, ^8^ with the majority of the Perth/WA samples falling into the C/Sao Paulo/378/82‐like clade (23/29; 79%) with the remainder (6/29; 21%) falling into the C/Kanagawa/1/76‐like clade, while the Victorian samples were evenly distributed in both of these clades (Fig. 1). The bootstrap values for all of these clade assignments were high. The WA and Victorian viruses generally grouped most closely with other viruses collected during the same time frame and showed little drift from other influenza C viruses collected elsewhere from earlier time periods.

## 4 Discussion

This is the first report characterizing influenza C viruses obtained from Australia. Influenza C viruses were detected sporadically in two widely separated states of Australia (Victoria and Western Australia) mainly in the major cities of these states (Melbourne and Perth which are 3420 km apart) during the period 2008–2014. The incidence of influenza C appeared to be low in Australia especially from the surveillance performed by the Sentinel GP network (SPNWA) in WA where over a 10‐year period only 13 cases of influenza C were detected with a maximum of six cases in 1 year representing only 0.8% of cases swabbed for ILI. Higher levels of infection were detected in the WAIVE program where children ≤ 5 years of age were enrolled. Detection levels in respiratory samples from swabs taken from these children with ILI symptoms reached as high as 5.8% positive for influenza C in 2014 and occurred at the same time as seasonal influenza A (both A(H3N2) and A(H1N1)pdm09) and influenza B circulated. The majority of these 2014 influenza C cases also had other pathogens identified in the same respiratory sample by real‐time PCR such as RSV, enteroviruses/rhinoviruses, or influenza A with only 4/31 cases (13%) having only influenza C alone detected. A study of young Nigerian children [5] found fewer coinfections with 8/12 (67%) of children having influenza C infections alone and two having coinfections with influenza C and adenovirus or other coinfections with enterovirus (1) or rhinovirus (1). A similar finding was reported in children (0.4–2.9 years) in the Philippines, where 7/15 cases (47%) with severe pneumonia were identified as having only influenza C present in their clinical respiratory samples, with six of them being admitted to hospital^8^; however, this study was based on the identification of coinfections by virus culture and may have therefore underestimated the true level of viral coinfections present, especially rhinoviruses. The high proportion of influenza C specimens in Australian children that were also RT‐PCR positive for other respiratory pathogens in the same clinical samples makes it difficult to determine the true role of influenza C in causing these children's illnesses. Interestingly the rate of admission to hospital from those cases with influenza C infections (and possibly with other respiratory viruses) was similar to the overall rate of hospital admissions in the WAIVE study, 11.1% vs 16.8%, respectively. The frequency of large influenza C epidemics in Australia is unknown, but looking at the WAIVE data (Table 2) there is some indication that they may occur approximately every 2 years, as the influenza C cases detected were the highest in 2010, 2012, and 2014. In Japan, it also appears that influenza C epidemics could be detected every couple of years.^14^

Overall, we were moderately successful in isolating influenza C, with 51.5% of original clinical samples yielding isolates. These isolates had Ct values in RT‐PCR assays (detecting the HE gene), ranging from 19 to 30 with similar results for the Perth 2014 samples (45.2% isolation rate). Influenza C has been known to be quite difficult to culture,^2^, ^15^ and some have suggested this may be one of the reasons that it is relatively understudied. In our study, some clinical samples that initially had influenza A and C detected by real‐time PCR, only yielded influenza A virus isolates even though the isolation was performed at 33°C (optimal for influenza C) instead of the usual 35°C (optimal for human influenza A).

The HE protein of influenza C plays a unique role in the influenza C life cycle being responsible for the receptor binding, the receptor destroying enzyme action, and the membrane fusion activities of the virus.^16^ A phylogenetic analysis of the HE genes of the Australian influenza C sequences showed that of the six reference groups^17^, the Australian viruses only fell into two of these groups with the majority grouping into the C/Sao Paulo/378/82 clade and a minority of viruses grouping with C/Kanagawa/1/76 clade. Viruses from both Perth and Victoria were found in both of these clades with the viruses from each state often occurring within the same branch and closely grouping according to the year of their collection. The finding of viruses collected in 2008 closely aligning with viruses from 2014 demonstrates the low rate of mutation of the HE gene in influenza C compared with the HA and NA of influenza A and B. A recent report by Odagiri et al.^8^ 2015 on influenza C viruses collected in Japan and the Philippines from 2008 to 2013 found that all 19 influenza C viruses sequenced, belonged to the C/Sao Paulo/378/82‐lineage. Interestingly, a study of circulating influenza C viruses in 2014 in Yamagata, Japan^17^, found both C/Kanegawa/1/76‐lineage and C/Sao Paulo/378/82‐lineage influenza C viruses in circulation with the C/Kanegawa/1/76‐lineage predominating most recently. Pabbaraju et al.^1^ 2013 also found cocirculation of influenza C viruses from these two clades during in a Canadian study conducted with samples collected between 2010 and 2012 from children of less than 10 years of age. In a Paediatric Clinic of the University of Milan, a study over 4 years (2008–9 to 2011–2) in children under 15 years of age with influenza C and radiographically confirmed community‐acquired pneumonia, five cases were identified with two viruses of the C/Kanagawa/1/76‐lineage and three from the C/Sao Paulo/378/82‐lineage.^18^ Clearly, viruses from these two influenza C lineages have been the dominant clades seen in many countries in recent years even though they appear to have changed very little based on their HE phylogeny, since they were first reported.

Unfortunately, our sample size was not large enough to be able to gain an in depth understanding of influenza C within Australia. Nor can it determine the level of reassortment that has occurred as the six internal genes have yet to be sequenced. However, Odagiri et al.^8^ 2015 reported that although extensive reassortment was observed with C/Sao Paulo/378/82‐lineage viruses, these viruses remained antigenically closely related. The true impact of influenza C infections in children may well vary with age, ethnicity, health status, and the presence of other etiologic bacteria and viruses and may be influenced by the irregular occurrence of epidemics in different geographic locations. While many influenza C cases are asymptomatic and have minimal impact on health services, periodically larger outbreaks occur – such as the one that was seen in Perth in 2014 – which may infect substantial numbers of children and possibly immunocompromised or vulnerable populations, which is likely to result in significant morbidity with increased hospital visits and admissions. Hence, influenza C screening should be considered for addition to respiratory screening panels, preferably using real‐time PCR or another equally sensitive test, to determine the full impact of this virus on the population, especially young children.

## References

[1] Pabbaraju K , Wong S , Wong A , May‐Hadford J , Tellier R , Fonseca K . Detection of influenza C virus by real time RT‐PCR assay. Influenza Other Respir Viruses. 2013;7:954–960. 23445084 10.1111/irv.12099 PMC4634283

[2] Matsuzaki Y , Sugawara K , Abiko C , et al. Epidemiological information regarding the periodic epidemics of influenza C virus in Japan (1996‐2013) and the seroprevalence of antibodies to different antigenic groups. J Clin Virol. 2014;61:87–93. 25017953 10.1016/j.jcv.2014.06.017

[3] Calvo C , García‐García ML , Borrell B , Pozo F , Casas I . Prospective study of influenza C in hospitalized children. Pediatr Infect Dis J. 2013;32:916–919. 23624431 10.1097/INF.0b013e31828fca10

[4] Smith DB , Gaunt ER , Digard P , Templeton K , Simmonds P . Detection of influenza C virus but not influenza D virus in Scottish respiratory samples. J Clin Virol. 2016;74:50–53. 26655269 10.1016/j.jcv.2015.11.036 PMC4710576

[5] Akinloye OM , Rönkkö E , Savolainen‐Kopra C , , et al. Specific viruses detected in nigerian children in association with acute respiratory disease. J Trop Med. 2011;2011:690286. 22007241 10.1155/2011/690286 PMC3191740

[6] Kauppila J , Rönkkö E , Juvonen R , et al. Influenza C virus infection in military recruits–symptoms and clinical manifestation. J Med Virol. 2014;86:879–885. 24122799 10.1002/jmv.23756

[7] Shimizu Y , Abiko C , Ikeda T , Mizuta K , Matsuzaki Y . Influenza C virus and human metapneumovirus infections in hospitalized children with lower respiratory tract illness. Pediatr Infect Dis J. 2015;34:1273–1275. 26244834 10.1097/INF.0000000000000863

[8] Odagiri T , Matsuzaki Y , Okamoto M , et al. Isolation and characterization of influenza C viruses in the Philippines and Japan. J Clin Microbiol. 2015;53:847–858. 25552361 10.1128/JCM.02628-14 PMC4390655

[9] Salez N , Mélade J , Pascalis H , et al. Influenza C virus high seroprevalence rates observed in 3 different population groups. J Infect. 2014;69:182–189. 24704348 10.1016/j.jinf.2014.03.016

[10] Sentinel Practitioners Network of Western Australia – SPN (WA). See http://ww2.health.wa.gov.au/Articles/F_I/Infectious-disease-data/Sentinel-Practitioners-Network-of-Western-Australia-SPNWA. Accessed 15 February 2016.

[11] Blyth CC , Jacoby P , Effler PV , et al. Effectiveness of trivalent flu vaccine in healthy young children. Pediatrics. 2014;133:e1218–e1225. 24753525 10.1542/peds.2013-3707

[12] Chakraverty P . The detection and multiplication of influenza C virus in tissue culture. J Gen Virol. 1974;25:421–425. 4475095 10.1099/0022-1317-25-3-421

[13] Guindon S , Dufayard JF , Lefort V , Anisimova M , Hordijk W , Gascuel O . New algorithms and methods to estimate maximum‐likelihood phylogenies: assessing the performance of PhyML 3.0. Syst Biol. 2010;59:307–321. 20525638 10.1093/sysbio/syq010

[14] Tanaka S , Aoki Y , Matoba Y , et al. The dominant antigenic group of influenza C infections changed from c/Sao Paulo/378/82‐lineage to c/Kanagawa/1/76‐lineage in Yamagata, Japan, in 2014. Jpn J Infect Dis. 2015;68:166–168. 25797148 10.7883/yoken.JJID.2014.520

[15] Racaniello VR , Palese P . Isolation of influenza C virus recombinants. J Virol. 1979;32:1006–1014. 513198 10.1128/jvi.32.3.1006-1014.1979 PMC525950

[16] Wang M , Veit M . Hemagglutinin‐esterase‐fusion (HE) protein of influenza C virus. Protein Cell. 2016;7:28–45. 26215728 10.1007/s13238-015-0193-x PMC4707155

[17] Yano T , Maeda C , Akachi S , et al. Phylogenetic analysis and seroprevalence of influenza C virus in Mie Prefecture, Japan in 2012. Jpn J Infect Dis. 2014;67:127–131. 24647258 10.7883/yoken.67.127

[18] Principi N , Scala A , Daleno C , Esposito S . Influenza C virus–associated community‐acquired pneumonia in children. Influenza Other Respir Viruses. 2013;7:999–1003. 23594251 10.1111/irv.12062 PMC4634288

