# Supplementary figures and images for: Influenza C infections in Western Australia and Victoria from 2008 to 2014
Source: Influenza Other Respir Viruses. 2016 Jul 23;10(6):455–61. doi: 10.1111/irv.12402 (PMC5059950; doi:10.1111/irv.12402)

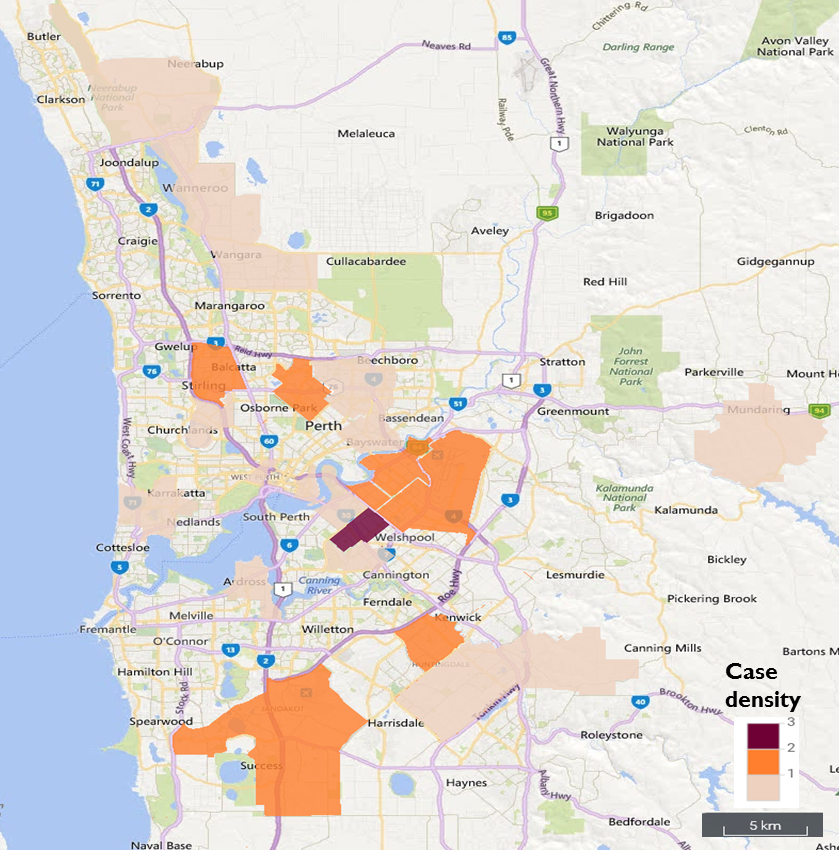

Supplement: Supplementary file 1 [file IRV-10-455-s001.tif]
