# Supplementary material for: Influenza C infections in Western Australia and Victoria from 2008 to 2014
Source: Influenza Other Respir Viruses. 2016 Jul 23;10(6):455–61. doi: 10.1111/irv.12402 (PMC5059950; doi:10.1111/irv.12402)
Supplement: Supplementary file 2 [file IRV-10-455-s002.docx]

Table S1

|  | **Designation** | **GISAID accession no.** |
| --- | --- | --- |
| 1 | C/PERTH/1/2014 | EPI_ISL_202517 |
| 2 | C/PERTH/2/2014 | EPI_ISL_202518 |
| 3 | C/PERTH/5/2014 | EPI_ISL_202519 |
| 4 | C/PERTH/8/2014 | EPI_ISL_202520 |
| 5 | C/PERTH/9/2014 | EPI_ISL_202512 |
| 6 | C/PERTH/10/2014 | EPI_ISL_202513 |
| 7 | C/PERTH/12/2014 | EPI_ISL_202514 |
| 8 | C/PERTH/15/2014 | EPI_ISL_202523 |
| 9 | C/PERTH/17/2014 | EPI_ISL_202521 |
| 10 | C/PERTH/19/2014 | EPI_ISL_202522 |
| 11 | C/PERTH/23/2014 | EPI_ISL_202515 |
| 12 | C/PERTH/26/2014 | EPI_ISL_202516 |
| 13 | C/PERTH/31/2014 | EPI_ISL_202511 |
| 14 | C/PERTH/1/2012 | EPI_ISL_212070 |
| 15 | C/PERTH/2/2012 | EPI_ISL_212071 |
| 16 | C/PERTH/3/2012 | EPI_ISL_212072 |
| 17 | C/PERTH/5/2012 | EPI_ISL_212073 |
| 18 | C/PERTH/6/2012 | EPI_ISL_212074 |
| 19 | C/PERTH/7/2012 | EPI_ISL_212075 |
| 20 | C/PERTH/8/2012 | EPI_ISL_212076 |
| 21 | C/PERTH/9/2012 | EPI_ISL_212077 |
| 22 | C/PERTH/10/2012 | EPI_ISL_212078 |
| 23 | C/PERTH/11/2012 | EPI_ISL_212079 |
| 24 | C/PERTH/12/2012 | EPI_ISL_212080 |
| 25 | C/PERTH/13/2012 | EPI_ISL_212081 |
| 26 | C/PERTH/1/2008 | EPI_ISL_212084 |
| 27 | C/PERTH/2/2008 | EPI_ISL_212083 |
| 28 | C/VICTORIA/2/2014 | EPI_ISL_212088 |
| 29 | C/VICTORIA/3/2014 | EPI_ISL_212089 |
| 30 | C/VICTORIA/4/2014 | EPI_ISL_212090 |
| 31 | C/VICTORIA/2/2012 | EPI_ISL_212086 |
| 32 | C/VICTORIA/4/2012 | EPI_ISL_212087 |
| 33 | C/VICTORIA/1/2011 | EPI_ISL_118655 |
| 34 | C/PERTH/9/2010 | EPI_ISL_212082 |
| 35 | C/PERTH/3/2008 | EPI_ISL_212085 |
